# Foreign body removal from nasal cavity in transorbital injury of long bamboo stick penetrating nasopharyngeal region: a case report

**DOI:** 10.3389/fsurg.2026.1779911

**Published:** 2026-03-10

**Authors:** Duo Yang, Su-Xia Peng, Jun Li, Man-Ting Yi, Hao Liang

**Affiliations:** 1 Department of Ophthalmology III, Jili Hospital of Liuyang City, Liuyang, Hunan, China; 2 Institute of TCM Diagnostics, Hunan University of Chinese Medicine, Changsha, Hunan, China

**Keywords:** case report, multidisciplinary surgical approach, nasopharyngoscopy, plant-based foreign body, transorbital penetrating injuries

## Abstract

Transorbital penetrating injuries (TPI) are rare yet potentially life-threatening, requiring prompt and precise surgical management. This case report highlights the complex management of a 67-year-old male with a transorbital injury caused by a bamboo stick penetrating into the nasopharynx. The patient presented with a transorbital injury initially misdiagnosed as “gas formation” on CT imaging. Multi-angle CT scans later identified the full extent of the foreign body. A multidisciplinary team planned a nasal cavity approach to access and remove the foreign body. The bamboo stick was transected within the nasal cavity and extracted with minimal tissue damage. Postoperatively, the patient recovered rapidly, with improved visual acuity and slightly limited eye movement. This case underscores the importance of accurate imaging, timely multidisciplinary intervention, and a tailored surgical approach in managing complex TPI to optimize outcomes.

## 1 Introduction

Transorbital penetrating injuries (TPI) represent a rare form of cranial trauma, necessitating immediate, coordinated surgical responses. Given orbit proximity to the cranial cavity and paranasal sinuses, penetrating injuries to the orbit can lead to significant complications, including damage to ocular structures, intracranial penetration, or injury to the sinuses, which can culminate in severe morbidity or mortality (1). Unlike inorganic materials (e.g., metallic and plastic), which primarily cause mechanical damage, plant-based foreign bodies can provoke additional complications, such as infections and chronic inflammation, necessitating prompt and thorough removal (2). Moreover, wooden foreign bodies also bring diagnostic challenges due to their low density (3), which may remain undetected or misdiagnosed because they can mimic air, fat, or muscle within the orbit.

This case report presents a rare instance of a long bamboo stick penetrating the orbit and extending into the nasopharynx, highlighting the complexities of managing large, organic foreign bodies in transorbital injuries. The surgical approach to such cases must be meticulously planned, considering the foreign body's size, trajectory, and potential for collateral damage during extraction. We discuss the diagnostic pitfalls encountered, detail the rationale behind the selection of an endoscopic nasal approach over traditional orbital or nasopharyngeal routes, and summarize the key lessons learned to guide the management of similar complex TPI cases. This report was prepared following the CARE Guidelines.

## 2 Case description

### 2.1 History

**Figure 1:**
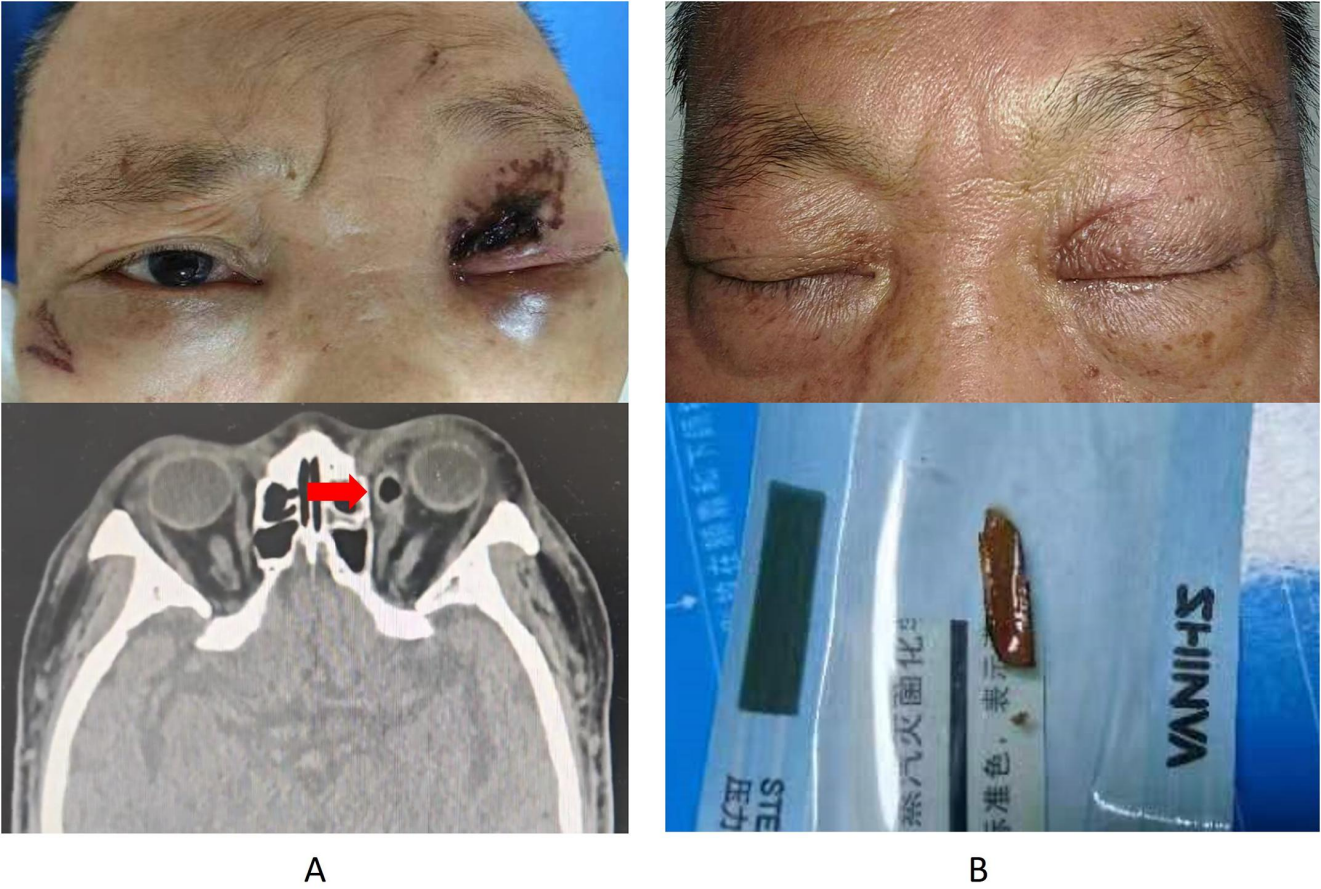
Clinical imaging and photos of first surgery. **(A)** Photograph showing Left eye exhibited redness and swelling and computed tomograph showing “gas formation”. **(B)** Photograph of wound healing and wooden fragment of foreign body. Red Arrow: gas formation.

On an April evening in 2024, a 67-year-old male sustained a fall, landing face-first, and felt a “small” object penetrate into his left eye. He immediately experienced pain, bleeding, and blurred vision and was admitted to a local hospital. The visual acuity of the left eye was 1.3 (logMAR). A CT scan revealed left facial soft tissue injury with pathological gas formation, contusions of the left medial rectus and eyeball, fractures of the left maxillary sinus roof and medial orbital wall, and hematocele in the left maxillary and ethmoid sinuses. He was transferred to Jili Hospital for surgery, during which debridement exposed a 5 mm deep wound, and a wooden fragment was removed. Post-suturing, the wound healed well without infection or exudates (Figure 1). The patient had no history of chronic diseases such as diabetes mellitus, hypertension, or angina. He was not on any regular medications and had no known allergies. He was a non-smoker and did not consume alcohol regularly. There was no family history of systemic illnesses or inherited ocular conditions.

**Figure 2:**
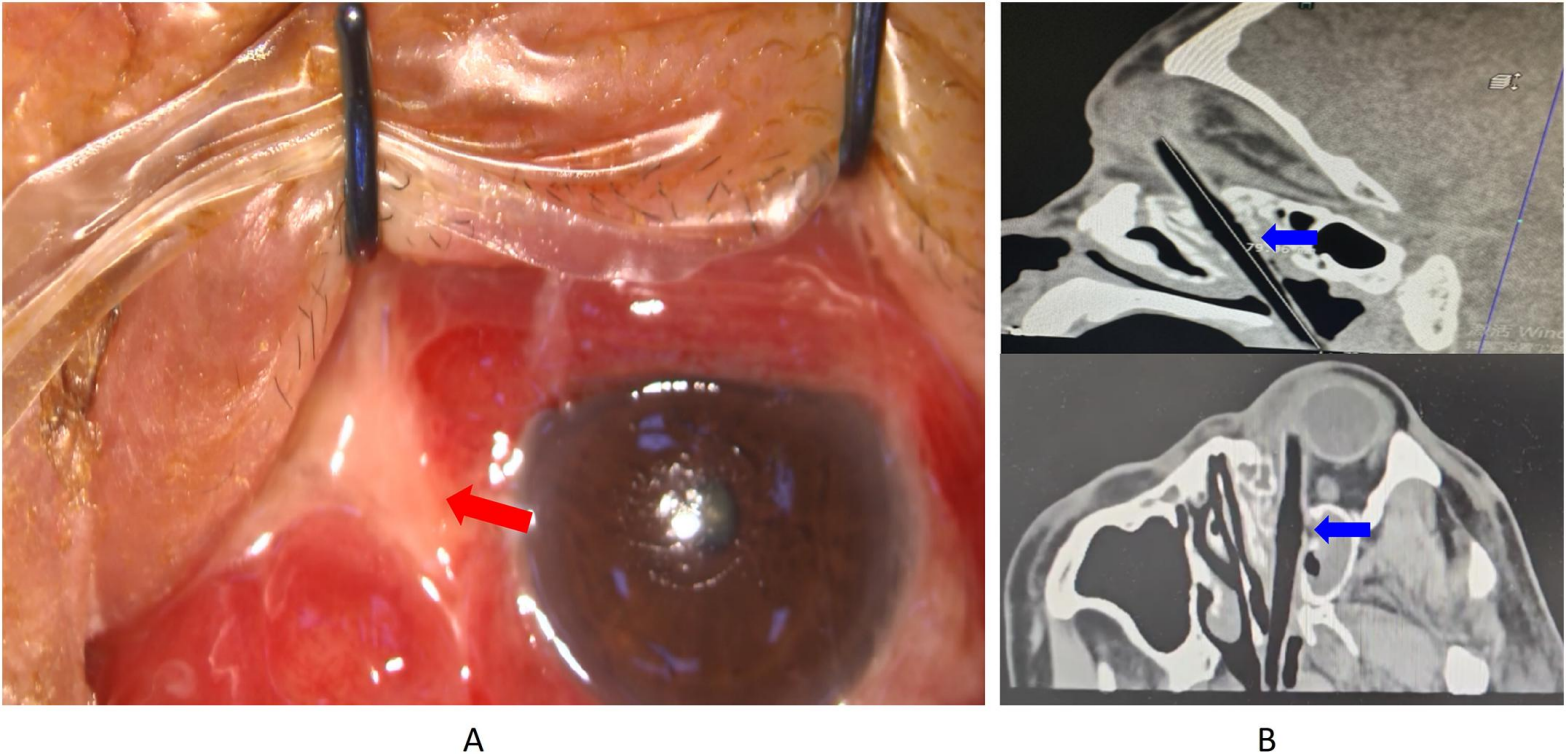
Clinical imaging and photos before the second surgery. **(A)** purulent discharge from the conjunctiva at the medial canthus, along with conjunctival edema and dissolution. **(B)** Computed tomograph of oblique sagittal, oblique coronal, and oblique horizontal planes suggesting the complete foreign body. Red Arrow: purulent discharge and conjunctival edema. Blue Arrow: Imaging of the complete foreign body.

During hospitalization, persistent purulent discharge from the conjunctiva at the medial canthus, conjunctival edema, and limited inward eyeball movement were observed. The sclera was obscured, while the cornea and lens remained clear. Fundus imaging showed a visible optic disc with clear boundaries, a C/D ratio of 0.3, an A/V ratio of 2:3, and a macular central reflex. Five days post-injury, a repeat orbital CT scan revealed “gas” accumulation from the temporal side of the left medial rectus toward the nasal cavity, accompanied by bone destruction. This suggested a penetrating low-density object. Additional CT imaging in oblique sagittal, coronal, and horizontal planes identified a foreign body entering through the medial canthus, adhering to the eyeball wall, and penetrating the nasopharynx. Nasopharyngoscopy confirmed the presence of a bamboo fragment on the posterior pharyngeal wall (Figure 2). The timeline of the patient is displayed in Supplementary Figure S1.

### 2.2 Surgery

A multidisciplinary treatment (MDT) team consisting of neurosurgeons, ophthalmologists, and otolaryngologists convened to collaboratively develop surgical planning for the patient. The CT imaging indicated that the foreign body was a thicker middle section with tapered ends, as it was relatively slender in the orbital segment, had the largest diameter within the nasal cavity, and became relatively thin again in the nasopharyngeal segment. Within 5 days post-injury, some tissues began to heal and encapsulate the foreign body. Three potential surgical approaches for removing the foreign body are initially considered: extraction via the orbital route, extraction from the tip of the foreign body in the nasopharynx, and via the nasal cavity. We finally concluded that removing the foreign body through the nasal cavity would be the safest approach.

**Figure 3:**
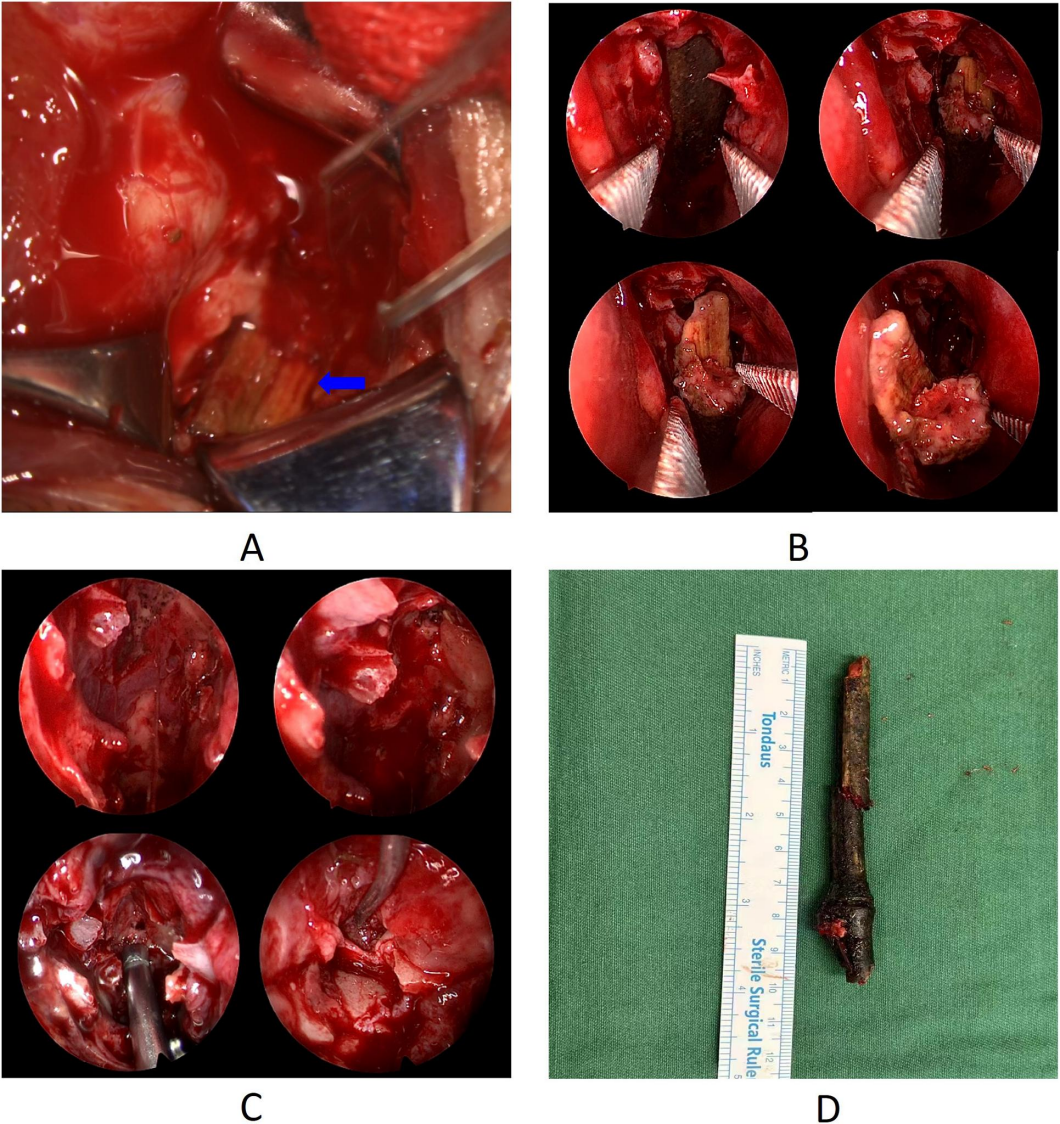
Endoscopic removal of the foreign body. **(A)** Exploration of the orbit finding the tip of the wooden foreign body. **(B)** Endoscopic removal of the foreign body from the nasal cavity. **(C)** Cleaning the fragments in the ethmoid sinus and nasal cavity. **(D)** length of the main body of the extracted foreign body. Blue Arrow: The tip of the wooden foreign body in orbit.

Endoscopic removal of the foreign body from the nasal cavity was performed under general anesthesia. The surgeons first explored the orbit at the site of the conjunctival scar in the medial canthus and found that the tip of the wooden foreign body was tightly adherent to the wall of the eyeball (Figure 3A). Attempts to move the foreign body were unsuccessful as it was firmly lodged. The endoscopic examination was then performed through the left nostril, revealing that the midsection of the foreign body was lodged in the nasal cavity, partially obscured by the middle turbinate's uncinate process. The uncinate process of the ethmoid bone was excised with a hook knife, and the middle turbinate was retracted, displaying that the foreign body had penetrated the nasal cavity through the middle meatus and was directed towards the nasopharynx via the inferior meatus. Bone forceps were used to sever the root of the foreign body, removing the portion located in the nasopharynx and creating space within the nasal cavity. Following the long axis of the foreign body, a vascular clamp was gently used to pull the foreign body downward, allowing the distal tip to detach from the orbital cavity (Figure 3B). Due to the limited space, the bone forceps were again used to sever the remaining portion of the foreign body root, until the tip was exposed enough within the nasal cavity, and then the main body of the object was removed accordingly (Figure 3B). Upon examining the path of the foreign body, it was found to traverse the left ethmoid sinus, where parts of the sinus cavities were damaged, and an amount of foreign body fragments remained. With the aid of a 70° nasal endoscope, the fragments within the ethmoid sinus were carefully removed, and hemostasis was achieved using a piece of gel foam (Figure 3C). After exploring the orbit of the left eye again, it was observed that the foreign body had been completely removed. The foreign body was a long bamboo stick, and even excluding the severed portions, its length of the main body was nearly 10 cm (Figure 3D). The Partial muscle fiber rupture and dissolution were noted in the medial rectus, with scleral lesion beneath the tendon of the medial rectus, exposing a small area of black choroid. Following extensive irrigation with normal saline, the areas of dissolved sclera were intermittently sutured using 8-0 absorbable sutures. The medial rectus muscle belly and then the conjunctival tissue were intermittently sutured. The surgery was finished, with intraoperative bleeding of 10 mL from the nasal cavity and 2 mL from the orbit.

### 2.3 Follow up

**Figure 4:**
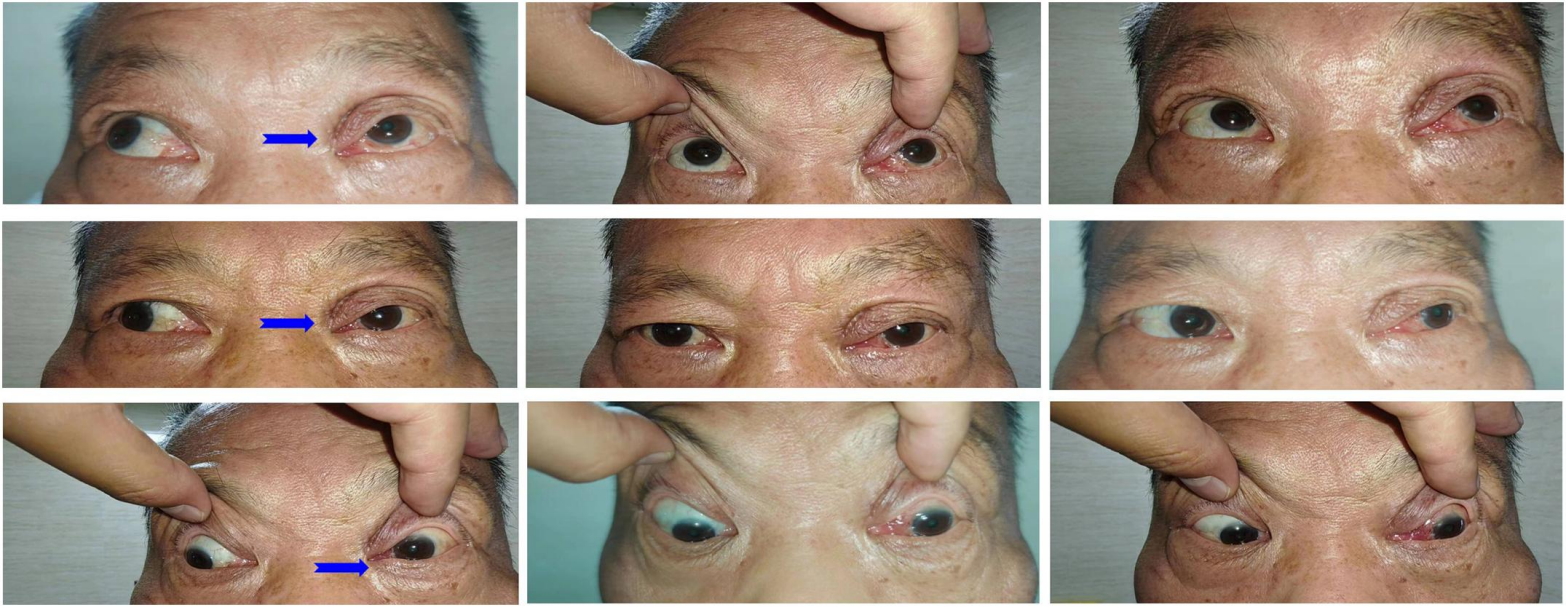
Follow up examination of eye movement. Blue Arrow: Limited left eye adduction.

The patient was treated postoperatively with cefuroxime for infection prevention, dexamethasone for inflammation, and phenylephrine for hemostasis. The recovery was satisfactory. After 3-month follow up, visual acuity of left eye improved from 1.3 to 0.5 (logMAR), with intraocular pressure around 15 mmHg. Slightly limited left eye adduction was observed (Figure 4); although visual acuity in the left eye remained significantly lower than in the right, the patient reported no noticeable diplopia after compensating with head positioning. Fundus examination revealed a retinal scar approximately 3 disc diameters in size on the nasal side, while the macula and optic disc structures appeared normal.

## 3 Discussion

There have been many cases published of unusual foreign bodies that have penetrated intracranially via the orbit (4). The orbit's pyramidal shape inherently directs penetrating objects toward critical areas (5, 6). They may even penetrate the dura and cause intracranial lodgings (1). Plant-based foreign bodies are typically non-manufactured objects with irregular shapes, often causing more severe mechanical damage compared to other types of foreign bodies (7). These materials are prone to decay and have a porous texture that can harbor pathogenic microorganisms, leading to a high risk of infection. Additionally, due to their brittle nature, they are prone to fragmentation (8). In this case, the patient's first surgery removed only a small fragment of the foreign body. Postoperatively, the patient still exhibited persistent purulent discharge from the conjunctiva at the medial canthus, along with conjunctival edema and dissolution, and limited inward movement of the eyeball. Therefore, there is suspicion that parts of the foreign body may still be retained within the patient's head.

CT offers a clear advantage over MRI for detecting and localizing nonmetallic orbital penetrating foreign bodies of all sizes, except for plastic (9). Organic and plastic objects can be challenging or impossible to detect using x-ray/CT (10–12). Our experience reinforces that when clinical suspicion persists despite negative or ambiguous initial imaging, multiplanar CT reconstructions (axial, coronal, sagittal) are indispensable. A continuous, linear low-density tract, as eventually identified in our patient, should be considered highly suggestive of a retained organic foreign body until proven otherwise. Detectability of foreign bodies strongly depends on material and imaging modality.

The decision to undertake surgical intervention for TPI requires a complex, multidisciplinary approach (13, 14). Several surgical approaches and techniques are utilized to address TPI (15, 16), each tailored to the specifics of the injury and the anatomical structures involved. The decision for an endoscopic nasal cavity approach was the cornerstone of management. A multidisciplinary team evaluated three potential routes: 1) Orbital Route: Risked severe secondary injury to the already compromised medial rectus, sclera, and optic nerve by retracting the foreign body along its entry path (17). 2) Nasopharyngeal Route: Was limited by the narrow space, proximity to the endotracheal tube, and the thicker mid-section of the foreign body being firmly lodged. 3) Nasal Cavity Route (Chosen): Allowed direct access to the foreign body's thickest segment within the ethmoid sinus. This provided adequate space for controlled sectioning under endoscopic visualization, minimizing fragmentation risk and avoiding re-entry into the fresh orbital wound.

After transecting the foreign body within the nasal cavity, it was removed with minimal damage, and the procedure confirmed that this was optimal. This approach avoided re-entry into the already compromised orbital structures, thereby minimizing the risk of further damage to the medial rectus, sclera, or optic nerve (18, 19). Furthermore, the nasal route offered sufficient space to perform controlled transection of the bamboo stick under endoscopic guidance, reducing the likelihood of fragmentation or embolization. In contrast, the orbital route would have necessitated reopening the recently sutured conjunctival wound and risked exacerbating existing ocular injuries. Similarly, the nasopharyngeal approach was deemed technically challenging due to the limited space and the presence of the endotracheal tube, increasing the risk of accidental displacement of the foreign body.

Following the surgery, the patient recovered rapidly; despite the injury to the medial rectus muscle and limited eye adduction, visual acuity restored, with no impact on quality of life, and no severe adverse outcomes such as enophthalmos. Although this exact clinical scenario has not been previously described in the literature, recent studies support the use of endoscopic techniques for managing penetrating orbital injuries, particularly those involving the paranasal sinuses or skull base (20–22). In our case, the nasal route allowed safe access to the central segment of the foreign body lodged in the ethmoid sinus without disturbing the recently repaired orbital wound.

## 4 Conclusions

This case report highlights the successful management of a rare transorbital penetrating injury involving a bamboo foreign body, emphasizing the importance of early diagnosis, imaging, and multidisciplinary surgical planning. The careful selection of a nasal cavity approach for foreign body removal minimized collateral damage and facilitated a favorable outcome. This case underscores the value of tailored surgical strategies in managing transorbital foreign body injuries and the need for thorough imaging to guide appropriate intervention.

## Data Availability

The original contributions presented in the study are included in the article/Supplementary Material, further inquiries can be directed to the corresponding author.

## Supplementary material

SUPPLEMENTARY FIGURE S1 Timeline of the patient.
