# Supplementary figures and images for: Foreign body removal from nasal cavity in transorbital injury of long bamboo stick penetrating nasopharyngeal region: a case report
Source: Front Surg. 2026 Mar 10;13:1779911. doi: 10.3389/fsurg.2026.1779911 (PMC13008974; doi:10.3389/fsurg.2026.1779911)

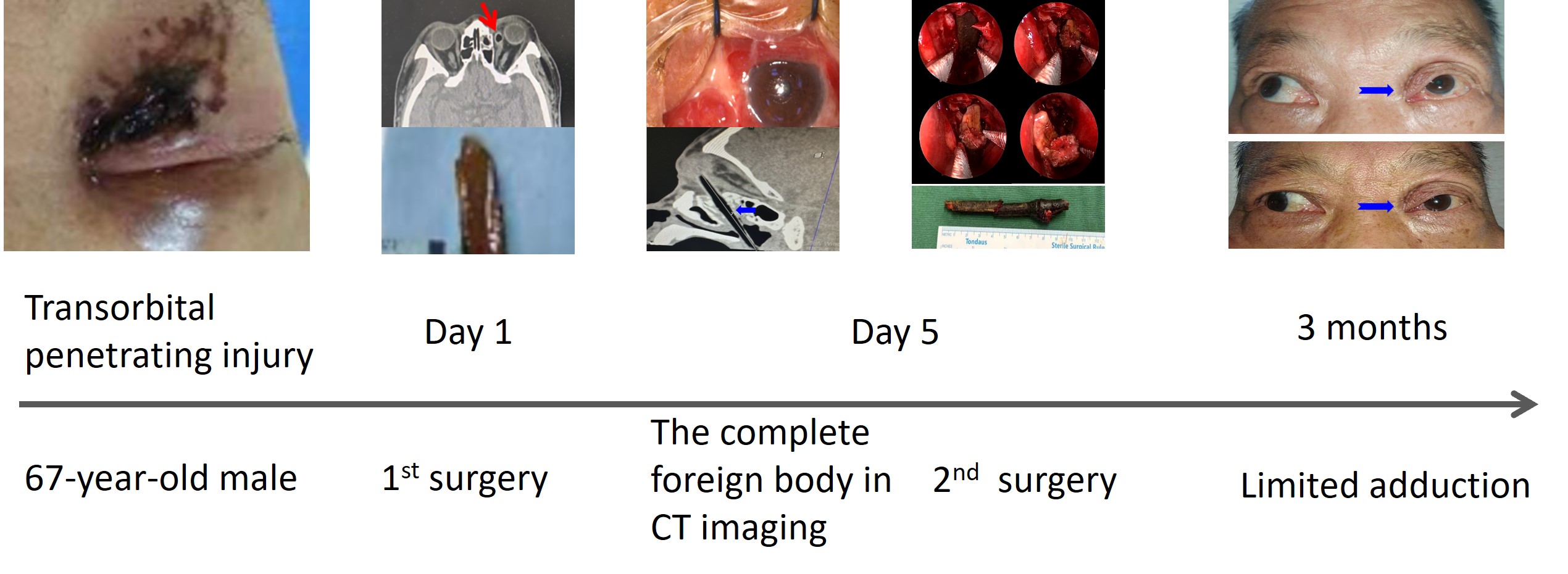

Supplement: SUPPLEMENTARY FIGURE S1 — Timeline of the patient. [file Image1.jpeg]
